# Effects of different nitrogen sources on the production of Hyaluronic acid by *Streptococcus*

**DOI:** 10.1186/1753-6561-8-S4-P204

**Published:** 2014-10-01

**Authors:** Nicole Pan, Renan Marques, Hanny Pereira, Josiane Vignoli, Maria Antonia Celligoi

**Affiliations:** 1 Universidade Estadual de Londrina, Londrina, PR, Brasil

## 

Hyaluronic acid (HA) is a linear polysaccharide with high molecular weight composed of disaccharide units of D-glucuronic acid (GlcUA) and N-acetylglucosamine (GlcNAc). HA is naturally present in vertebrate organisms, as well as in bacteria. HA can be obtained commercially through three routes: human umbilical cords, rooster combs, and strains of group C *Streptococcus*. This is a natural polysaccharide with extensive range of applications in the medical, pharmaceutical and cosmetics. Due to the viscoelastic and hydrophilic, the HA products and derivatives present high aggregated value ranging from US$ 2000 to 60000 Kg^-1^, depending on their applications. The most frequently used bacteria in the industrial production of HA are Lancefield group A and C streptococci. These bacteria are nutritionally fastidious microorganisms which require complex nutrients due to their limited ability to synthesize specific aminoacids and B-vitamins. Additionally, there is the nutritional requirement with respect to organic nitrogen, which also supplies a large portion of carbon for their cellular biosynthesis. Recent studies seek alternatives to allow the cost of production of HA using agricultural derivatives and industrial waste. In this context, the objective of this study was to evaluate the effect of nitrogen sources yeast extract, soy protein, whey protein and corn steep liquor in the production of HA. Fermentations were carried out in 125 mL erlenmeyer flasks containing 25 mL culture medium. The culture medium comprised in ( gL ^-1^): glucose, 30; nitrogen source (yeast extract, soy protein, whey protein or corn steep liquor), 30; K_2_HPO_4_, 2.5, NaCl, 2,0 and MgSO_4_, 1.5. The inoculum was 10% (v/v) and the fermentations occurred at 100 rpm, 37°C and pH 8.0 for 24 hours. The fermented medium was centrifuged and the cell free supernatant was treated with ethanol for the precipitation of HA which quantified using a colorimetric reagent Carbazole. The concentration of lactic acid, acetic acid and formic acid were also analyzed in a system of high performance liquid chromatography with IR detector, column Aminex HPX - 87H at 60°C and a solution of H_2_SO_4 _0.005 mol L^- 1 ^as the mobile phase a flow rate of 0.7 mL min^- 1^. The results performed in triplicate were compared by the Tukey test at 5% probability level (p < 0.05). The highest production of HA, 0.534 g L^-1 ^was obtained when using yeast extract as nitrogen source. Subsequently, experiments which resulted in a better yield of the polymer are those containing soybean protein (0.192 g.L^-1^) and whey protein ( 0.063 g.L^-1^). In medium containing corn steep liquor, no microbial growth or production of HA ocurred. The decreased production of HA was directly followed by a reduction of the production of lactic acid and acetic acid.

## References

[B1] Chong BF Nielsen LK Aerobic cultivation of Streptococcus zooepidemicus and the role of NADH oxidase Biochem Eng J 2003 16 153 162 10.1016/S1369-703X(03)00031-7

[B2] Chong BF Blanck LM Mclaughlin R Nielsen LK Microbial hyaluronic acid production Appl Microbiol Biotechnol 2005 66 4 341 351 10.1007/s00253-004-1774-4 15599518

[B3] Filisetti-Cozzi TM Carpita NC Measurement of uronic acids without interference from neutral sugars Anal Biochem 1991 197 1 157 62 10.1016/0003-2697(91)90372-Z 1952059

